# Effects of Hypoxia–Hyperoxia Preconditioning on Indicators of Muscle Damage After Acute Resistance Exercise in Male Athletes

**DOI:** 10.3389/fphys.2022.824210

**Published:** 2022-04-19

**Authors:** Peng-Wen Chen, Chi-Chieh Hsu, Li-Fan Lai, Chung-Pu Chi, Szu-Hsien Yu

**Affiliations:** ^1^Institute of Sports Sciences, University of Taipei, Taipei, Taiwan; ^2^Department of Aquatic Sports, University of Taipei, Taipei, Taiwan; ^3^Center for General Education, Taipei Medical University, Taipei, Taiwan; ^4^Department of Leisure Industry and Health Promotion, National Ilan University, Yilan City, Taiwan

**Keywords:** muscle soreness, maximal voluntary contraction, creatine kinase, muscle strength, inflammation

## Abstract

**Purpose:**

The purpose of this study was to investigate the effects of acute repeated hypoxia–hyperoxia preconditioning on resistance exercise (RE)-induced muscle damage in male athletes.

**Methods:**

Eleven young male athletes participated in this randomized double-blind counter-balanced crossover study, and were divided into Normoxia (N) and Hypoxia–Hyperoxia (HH) trials. Subjects of the respective trials were supplied with normoxic (FiO_2_ = 0.21), or alternating hypoxic/hyperoxic air (FiO_2_ = 0.10/0.99, 5 min each) for 60 min. Thirty minutes after preconditioning, subjects performed acute bouts of RE consisting of bench press, deadlift, and squats. Each exercise included 6 sets of 10 repetitions at 75% one-repetition maximum (1RM) with 2 min rest between sets. After a 2-week washout period, subjects changed trials and completed the same study procedure after the alternate preconditioning. Muscle soreness, maximal voluntary contraction (MVC), and circulating biochemical markers were tested before preconditioning (baseline) and during recovery at 0, 24, and 48 h after exercise.

**Results:**

Acute RE significantly increased levels of muscle soreness, creatine kinase (CK) and myoglobin (Mb), and decreased levels of peak knee extension torque in the N trial. Muscle soreness, CK, and Mb levels of the HH trial were significantly lower than that of the N trial after exercise. Interestingly, interleukin-6 (IL-6) levels of the HH trial increased significantly 0 h after exercise compared to baseline and were significantly higher than that of the N trial 0 and 24 h after exercise. However, no significant differences of thiobarbituric acid reactive substances (TBARS), cortisol, testosterone, peak torque, and average power levels were found between N and HH trials during recovery.

**Conclusion:**

Our data suggest that pre-exercise treatment of alternating hypoxic/hyperoxic air could attenuate muscle damage and pain after acute RE, but has no effect on muscle strength recovery in young male athletes.

## Introduction

In athletic training, resistance exercise (RE) effects are derived by controlling the loading, sets, repetitions, rest duration, and movement velocity of exercise, these stimulations result in physiological response and adaptation (Spiering et al., 2008). Heavy RE protocols that consist of high loads (<10 repetition maximum, RM), high volume (6 or more sets of 10 repetitions per exercise), short inter-set rests, and use of major muscle groups, are frequently used to induce muscle hypertrophy and improve strength in athletes (Tesch, 1988; Izquierdo et al., 2009). However, excess mechanical force during heavy RE protocols could injure muscle sarcomere, resulting in exercise-induced muscle damage (EIMD). Symptoms of delayed onset muscle soreness (DOMS) including increased muscle soreness and reduced muscle force appear during recovery periods 24–72 h after EIMD. In addition, because of increased cell membrane permeability and inflammatory response, levels of special muscular proteins and pro-inflammatory cytokines increase after EIMD (Smith, 1991; Cheung et al., 2003). Moreover, strenuous or unaccustomed exercise during training or competition also induce DOMS, negatively influencing exercise performance in athletes (Smith, 1992). Various protocols have been developed to alleviate DOMS induced by acute resistance or strenuous exercise, including the use of stretching (Dupuy et al., 2018), ice packs (Howatson and Van Someren, 2003), massage (Kargarfard et al., 2016), nutritional supplements (Harty et al., 2019) and anti-inflammatory drugs (Schoenfeld, 2012).

Systemic hypoxia–hyperoxia exposure is characterized by breathing several cycles of oxygen-depleted air (<20.9% inspired O_2_) followed by oxygen-enriched air (>20.9% inspired O_2_). Intermittent hypoxia–hyperoxia training (IHHT), resulting from chronic or prolonged hypoxia–hyperoxia exposure, has recently been found to positively impact a number of medical conditions. These include improving cardiorespiratory fitness (Dudnik et al., 2018); alleviating markers of diabetes and cognitive impairment (Serebrovska et al., 2019); and attenuating symptoms of coronary artery disease (Glazachev et al., 2017). In addition, IHHT has been used to accelerate the recovery of athletes with over training syndrome (Susta et al., 2017). The underlying physiological mechanism may be associated with upregulation of adaptive reactive oxygen species (ROS) signaling, because of increased ROS production during ischemic–reperfusion stress (Haffor and Al-Johany, 2005), resulting in better antioxidant capacity adaptation (Sazontova et al., 1994). However, the effects of acute systemic hypoxia–hyperoxia preconditioning on EIMD are still unclear.

Therefore, the main purpose of the present study was to investigate the effects of hypoxia–hyperoxia preconditioning on acute RE-induced muscle damage in male athletes. We hypothesized that acute hypoxia–hyperoxia preconditioning would attenuate the levels of muscle damage markers and improve the muscle function recovery following acute heavy RE in male athletes.

## Materials and Methods

### Subjects

Eleven male collegiate swimmers were recruited as subjects. Subjects belonged to competitive swimming teams at a physical education university and were trained professionally at least four times per week for more than 1 year on their respective university teams. The exclusion criteria for participation were as follows: smoking, cardiac arrhythmia, chronic metabolic diseases (diabetes, cardiovascular disease, or metabolic syndrome), and musculoskeletal injury. In order to reduce the metabolic effects associated with these substances, subjects were asked to avoid use of tobacco, alcohol, or sports supplements (whey protein, branch chain amino acids, or Chinese herbs etc.) from 1 month before to the end of the experiment. In addition, subjects maintained moderate physical activity and avoided high intensity or RE during the experiment. Subjects were informed of the purpose and experimental protocol of the study before experiments began, and they were informed of potential risks of participation and then signed their consent. The procedure of this study was ethically approved by the Institutional Review Board of University of Taipei, Taipei, Republic of China (Taiwan; Approved number: IRB-2016-052). Prior to the beginning of the study, demographic and anthropometric information including age, height, and weight was collected or measured by the research staff. Based on relevant investigations (Franz et al., 2018), *a priori* sample size of 11 subjects was calculated using the Sample Size Calculator.^1^ This group size was sufficient to detect a significant difference of CK levels between preconditioning and sham treatment groups 24 h after damaging exercise with *α* = 0.05 and *β* = 0.2.

### Study Design

In this randomized, double-blind, counter-balanced crossover study design, participants were randomly divided into Normoxia (N) or Hypoxia–Hyperoxia (HH) preconditioning trials. On the experiment day, subjects were supplied either normoxic air (FiO_2_ = 0.21) or alternated between hypoxic (FiO_2_ = 0.10) and hyperoxic (FiO_2_ = 0.99) air for 60 min according to their trial. Thirty minutes after N or HH preconditioning, subjects performed an acute bout of heavy RE to induce muscle damage. Muscle soreness and isokinetic muscle strength were determined before preconditioning and at 0, 24, and 48 h after exercise. Blood samples were collected at the same time points and used to assess the levels of muscle damage [creatine kinase (CK) and myoglobin (Mb)], inflammation (interleukin-6, IL-6), oxidative damage (thiobarbituric acid reactive substances, TBARS), and catabolism/anabolism (cortisol and testosterone). After the first trial, subjects switched preconditioning trials and completed the same study procedure. The trials were separated by a 2 weeks washout period. The study procedure was completed during off-season.

### Anthropometric and One-Repetition Maximum Measurements

One week before acute RE, subjects attended the laboratory for anthropometric (height and weight) measurements [mean height 172.5 ± 1.4 cm; median weight 70.3 (62.0–71.9); Table 1]. All subjects were then familiarized with the RE to be used in the study. After standardized 5 min light running and dynamic stretches, 1RM of each subject was determined for bench press, deadlift, and squat using the Smith machine (Cybex Plate Loaded 16,120 Smith Press, Cybex, Medway, MA, United States) according to a previous study (Maud and Foster, 2006). In order to ensure the correct motion and safety of subjects, 1RM testing and RE procedures in this study were supervised by a specialist. 1RM in squat, deadlift, and bench press were 99.1, 86.9, and 58.0 kg, respectively (Table 1).

**Table 1:** Physical characteristics of the subjects.

Variables	Values or numbers
N	11
Height (cm)	172.5 ± 1.4
Body weight (kg)	70.3 (62.0–71.9)
Age (years)	21.4 ± 0.3
BMI (kg/m^2^)	22.7 ± 0.5
1RM (kg)	
Squat	99.1 ± 4.5
Deadlift	86.9 ± 8.3
Bench press	58.0 ± 4.4

The values are mean ± SE or median (interquartile range). BMI, body mass index; 1RM, one-repetition maximum.

### Normoxia and Hypoxia–Hyperoxia Preconditioning

PVC balloons were filled with normoxic, hypoxic, or hyperoxic air, and supplied to subjects by mask and two-way non-rebreathing valves (T-shape 2,700, Hans Rudolph Inc., Kansas, MO, United States). Oxygen concentration of normoxic (FiO_2_ = 0.21), hypoxic (FiO_2_ = 0.10), or hyperoxic (FiO_2_ = 0.99) air was produced by mixing 100% oxygen and nitrogen gas, each resulting gas was then checked using Model GB300 Percent Portable Oxygen Analyzer (Teledyne Technologies Inc., City of Industry, CA, United States) before treatment. The HH preconditioning protocol consisted of 6 cycles of alternating hypoxia/hyperoxia (5 min each) for a total 60 min. In the HH trial, hypoxic and hyperoxic air were supplied, respectively, *via* Y tube and switched every 5 min using a switching valve. Normoxic air was continuously supplied in a blind manner to subjects of the N trial for 60 min also using the same type of Y tube in order to reduce the placebo effect.

### Acute Heavy Resistance Exercise Protocol

All resistance exercises in this study were completed using a Smith machine (Cybex Plate Loaded 16,120 Smith Press, Cybex, Medway, MA, United States). After standardized dynamic stretches, participants performed the squat, deadlift, and bench press for 10 repetitions at 50% of their 1RM to warm up before the acute RE protocol. This protocol was modified from a previous study (Vingren et al., 2009) and consisted of 3 training sessions of squat, deadlift, and bench press in order. Each session included 6 sets of 10 repetitions of each exercise at 75% 1RM with 2 min static rest between sets. Subjects were asked to performed full range of motion and maintain constant velocity during each motion. If subjects were unable to complete 10 repetitions in a set, additional 2 min static rest periods were implemented. Subjects were then allowed to complete any remaining repetitions.

### Isokinetic Muscle Strength and Muscle Endurance

The maximum voluntary contraction (MCV) of knee extensor was assessed using a Biodex System 4 isokinetic dynamometer (Biodex Medical Systems Inc., Shirley, NY, United States). Before testing, subjects performed a 5 min walking warm up on a treadmill. The right leg of each subject was investigated. Subjects were fixed in a rigid chair with 90° hip to knee angle, then performed 20 repetitions of knee extension and flexion between 90° and 0°. The angular velocity was fixed at 180° per second. Subjects exerted maximum power against the mechanism during extension and flexion. Knee extension peak torque (Newton-metre, N-m) and average power (Watts, W) were calculated as muscle strength and endurance, respectively.

### Muscle Soreness

Muscle soreness was scored using ratings of perceived muscle soreness (RPMS), a verbal rating scale (VRS) ranging from 0 to 6. The numbers corresponded with no soreness (0), dull feeling of soreness (1), light continuous soreness (2), more than light soreness (3), annoying soreness (4), severe soreness (5), and intolerable soreness (6; Rodenburg et al., 1993).

### Biochemical Analysis

Blood samples were taken using vacutainer tubes and needles (22-gauge) from the median antebrachial vein of the left forearm. After centrifugation at 3000 *g* and 4°C, supernatant (serum) was obtained and stored at −80°C until biochemical analysis was performed. A bench top DT-60II analyzer (Johnson and Johnson, NY, United States) was used to enzymatically analyze CK levels. Mb, IL-6, cortisol, and testosterone levels were tested using commercially available enzyme-linked immunosorbent assay (ELISA) kits. These kits were the Human Myoglobin ELISA Kit (Immunology Consultants Laboratory Inc., Portland, OR, United States), IL-6 ELISA Kit (Thermo Fisher Scientific Inc., Waltham, MA, United States), Cortisol ELISA (Immuno-Biological Laboratories Inc., Minneapolis, MN, United States) and Testosterone (Free) ELISA (Immuno-Biological Laboratories Inc., Minneapolis, MN, United States), respectively, and were used following the manufacturers’ protocols. The lipid peroxidation marker (malondialdehyde, MDA) was measured using a TBARS assay kit (Cayman Chemical Company, Ann Arbor, MI, United States).

### Statistical Analyses

All statistical analyses were performed using the SPSS 22.0 software (SPSS, Chicago, IL, United States). The data in figures and tables were expressed as changes from baseline. The Shapiro–Wilk normality test was used to analyze the normal distribution of all indicators. The normally and non-normally distributed variables are presented as mean ± standard error and 1st quartile median–3rd quartile, respectively. For non-normally distributed variables (soreness, CK, Mb, IL-6, testosterone, peak torque, and average power), the Friedman non-parametric statistical test was used to analyze the results at different time points within groups. The Wilcoxon signed rank test was used to determine the difference between N and HH trial results at the same time point. For normally distributed variables (TBARS and cortisol), two-way ANOVA with repeated measure was used to analyze mean differences. The results were considered to be significant when *p* < 0.05.

## Results

### Post-exercise Muscle Soreness Was Reduced by HH Preconditioning

Muscle soreness is shown in Table 2. In both trials, muscle soreness increased significantly (*p* < 0.05) 0 to 48 h after RE compared to baseline. The muscle soreness levels of the HH trial were significantly lower than those of the N trial at 24 h after RE.

**Table 2:** The muscle soreness measured before preconditioning and post-exercise in N and HH trials.

	Baseline	0 h	24 h	48 h
N	0.0 (0.0–0.0)	5.0 (4.0–5.0)^†^	4.5 (4.0–5.0)^†^	4.0 (3.0–4.0)^†^
HH	0.0 (0.0–0.0)	4.0 (4.0–4.0)^†^	4.0 (4.0–4.0)^†^^,^^*^	3 (3.0–3.5)^†^

The values are median (interquartile range; *n* = 11). N, normoxia; HH, hypoxia–hyperoxia.

† *p* < 0.05 vs. Baseline.

* *p* < 0.05 vs. N trial.

### Post-exercise Circulating Muscle Damage Markers Were Reduced by HH Preconditioning

**Figure 1:**
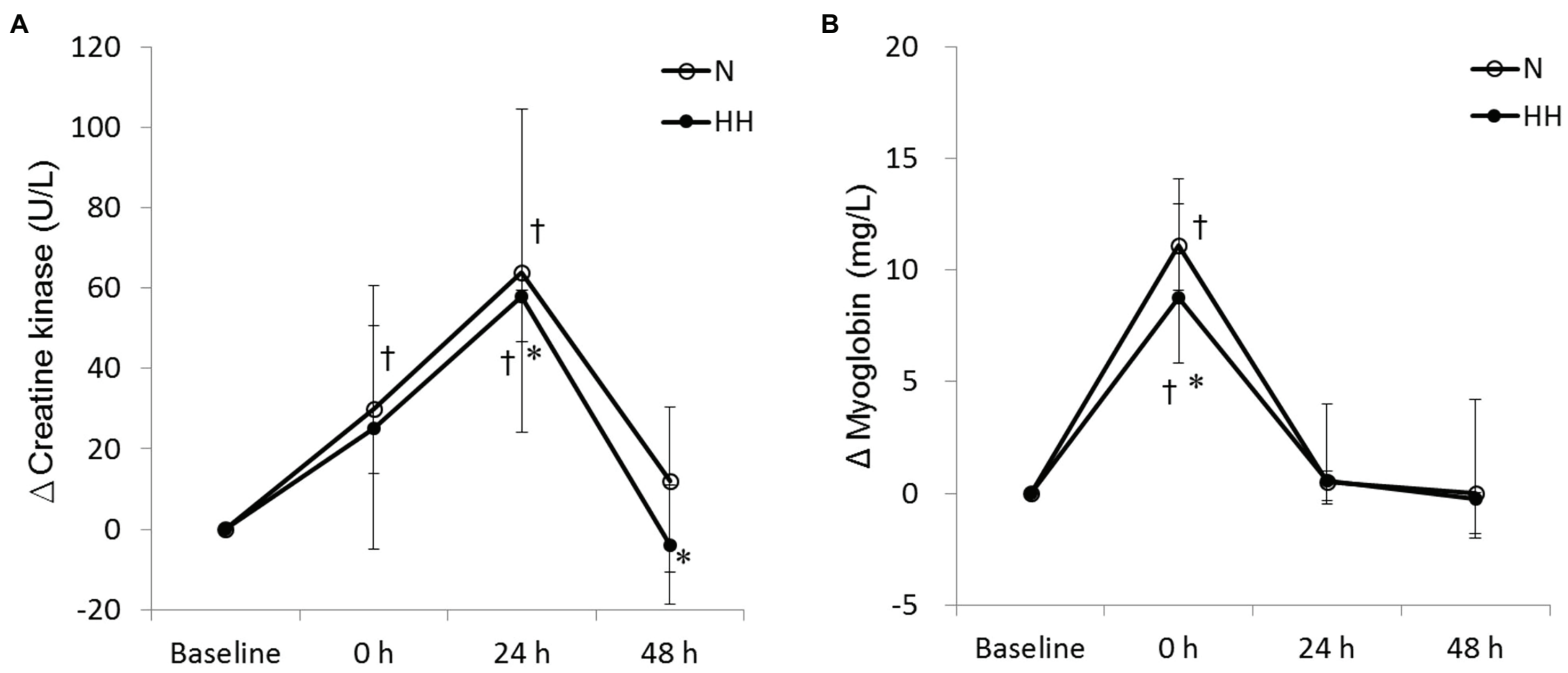
Changes in circulating creatine kinase **(A)** and myoglobin **(B)** levels before preconditioning and post-exercise in N and HH trials. The values are 1st quartile median–3rd quartile (*n* = 11). N, normoxia; HH, hypoxia–hyperoxia. ^†^*p* < 0.05 vs. Baseline. ^*^p < 0.05 vs. N trial.

**Figure 2:**
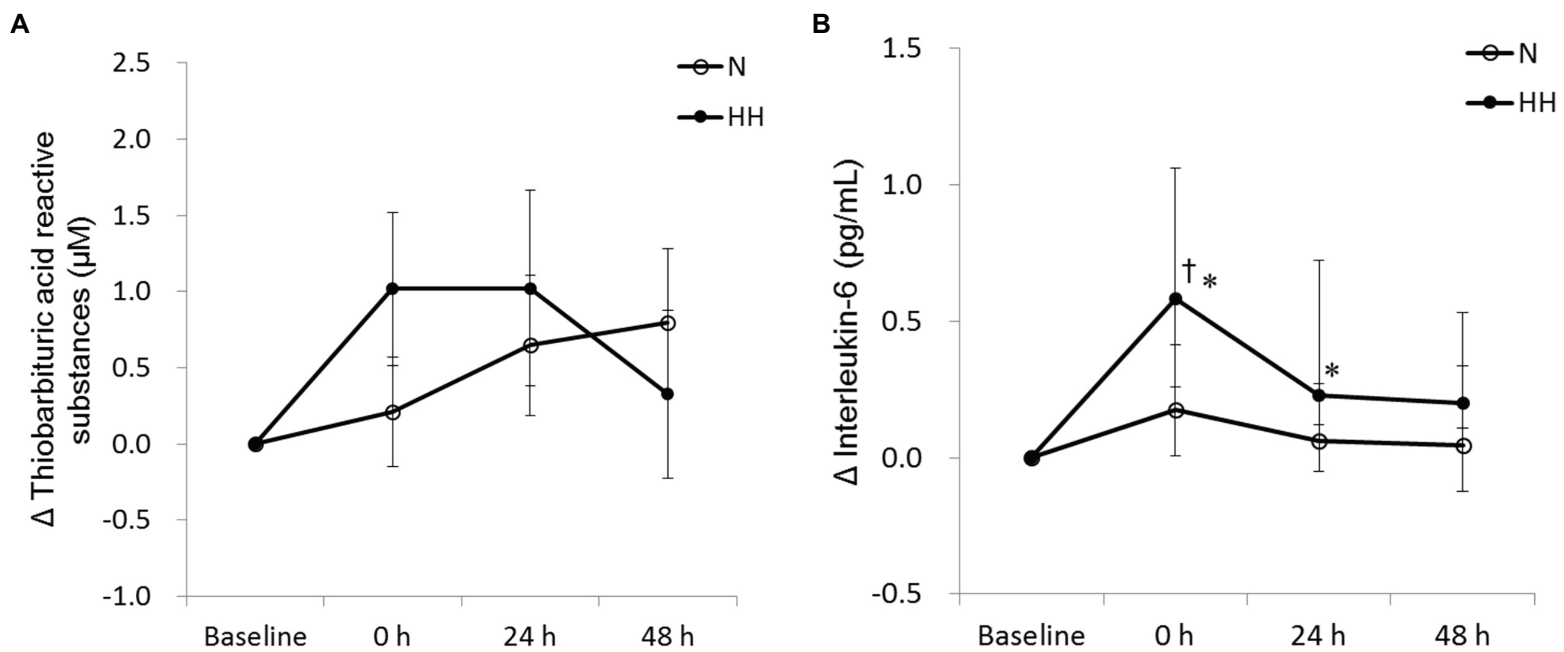
Changes in circulating thiobarbituric acid reactive substances (TBARS; **A**) and interleukin-6 **(B)** levels before preconditioning and post-exercise in N and HH trials. The values of TBARS and interleukin-6 are presented mean ± SE or 1st quartile median–3rd quartile, respectively (*n* = 11). N, normoxia; HH, hypoxia–hyperoxia. ^†^*p* < 0.05 vs. Baseline. ^*^p < 0.05 vs. N trial.

**Figure 3:**
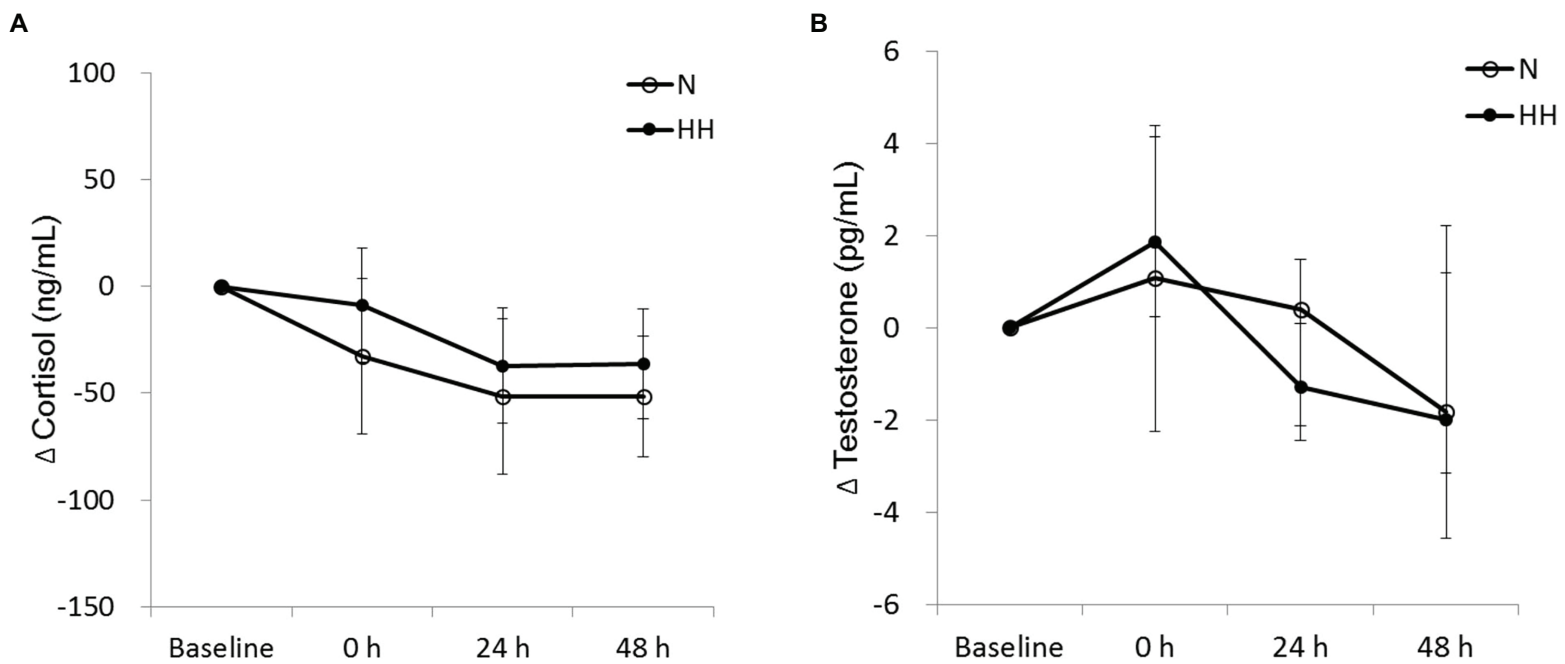
Changes in circulating cortisol **(A)** and testosterone **(B)** levels before preconditioning and post-exercise in N and HH trials. The values of cortisol and testosterone are presented mean ± SE or 1st quartile median–3rd quartile, respectively (*n* = 11). N, normoxia; HH, hypoxia–hyperoxia.

The circulating biochemical markers are shown in Figures 1–3. The CK levels were significantly increased at 0 and 24 h after RE in the N trial and only significantly increased at 24 h after RE in the HH trial. The CK levels of the HH trial were significantly lower than those of the N trial at 24 and 48 h after RE (Figure 1A). In both trials, the Mb levels increased immediately after RE and returned to baseline 24 to 48 h after RE. The Mb levels of the HH trial were significantly lower than those of the N trial at 0 h after RE (Figure 1B). RE did not increase TBARS levels significantly in both trials. There was no statistically significant TBARS level difference between trials at all time points (Figure 2A). The IL-6 levels only increased significantly from baseline in the HH trial at 0 h after RE and were significantly higher than those of the N trial at both 0 and 24 h (Figure 2B). Cortisol and testosterone levels did not significantly change after RE in both trials. No statistically significant differences of cortisol and testosterone were found between the N and HH trials at all time points (Figures 3A,B).

### HH Preconditioning Had no Effect on Isokinetic Muscle Strength and Muscle Endurance Following Exercise

**Figure 4:**
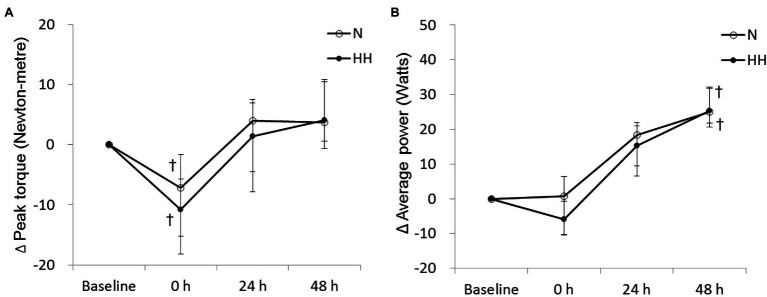
Changes in peak torque **(A)** and average power **(B)** levels during maximal voluntary contraction (MVC) test before preconditioning and post-exercise in N and HH trials. The values are 1st quartile median–3rd quartile (*n* = 11). N, normoxia; HH, hypoxia–hyperoxia. ^†^*p* < 0.05 vs. Baseline.

The peak torque and average power of knee extension are shown in Figure 4. Peak torque decreased significantly immediately after RE and returned to baseline 24 to 48 h after RE in both trials (Figure 4A). Average power increased significantly at 48 h after RE in both trials (Figure 4B). However, no significant difference was observed between trials at all time points (Figures 4A,B).

## Discussion

The biochemical and mechanical stresses caused by RE included inflammatory response, free radical generation, and muscle cell damage. These stresses temporarily cause reduced skeletal muscle function. To examine the potential of acute HH preconditioning to impact recovery from RE, we measured a number of markers including muscle soreness, cell damage, inflammation, oxidative damage, and isokinetic muscle function. The major findings of this study are as follows: (1) Acute HH preconditioning showed a protective effect resulting in reduced muscle damage after RE when compared to the N trial. This was evidenced by decreased circulating levels of CK and Mb, and reduced muscle soreness. (2) Interestingly, inflammatory cytokine levels after RE were increased with HH preconditioning, evidenced by elevation of IL-6 levels. (3) Catabolic, anabolic signal, and isokinetic muscle function response to RE were not affected by HH preconditioning. Taken together, our data suggest that the RE-induced muscle damage is partly blunted by HH preconditioning in young male athletes.

Levels of CK and Mb are important circulating indirect indicators of muscle damage response to strenuous or unaccustomed exercise (Sorichter et al., 1999). In agreement with previous RE studies (Bartolomei et al., 2017), increased levels of CK and Mb after RE protocols were observed in this study. In addition, RE-induced muscle damage levels were reduced by HH treatment according to CK and Mb data (Figure 1). A previous study reported that chronic HH treatment could reduce stress and damage under some conditions. Four days of IHHT pre-treatment could reduce serum troponin I levels after coronary artery bypass graft (CABG) surgery compared to sham treatment in patients with ischemic heart disease (Tuter et al., 2018). However, to the best of our knowledge, this is the first study using acute repeated HH treatment to blunt stress-induced physiological damage.

ROS plays an important role in muscle damage after exercise. However, the results demonstrated that circulating TBARS levels were not significantly elevated after RE in N trial (Figure 2A). This result is inconsistent with previous studies which reported elevated lipid peroxidation after RE (Güzel et al., 2007; Deminice et al., 2010). The inconsistent results may be explained by low sensitivity of TBARS assay (Frijhoff et al., 2015). In addition, 2 weeks of daily hypoxia (10% O_2_)–hyperoxia (30% O_2_) treatment can reduce acute hypoxia (Gonchar and Mankovska, 2012a) or Fe^2+^/ascorbate (Gonchar and Mankovska, 2012b)-induced lipid peroxidation. However, in this study, results showed that lipid peroxidation increased marginally (*p* = 0.071 by *t*-test) in the HH trial immediately after exercise, evidenced by increased TBARS levels after exercise (Figure 2A). It is possible that this TBARS response may be due to the stress induced by HH preconditioning, but without oxidative stress measurements during HH preconditioning this conclusion is uncertain.

Circulating IL-6 often increases after an acute bout of RE, which indicates an inflammatory response following exercise. In this study, circulating IL-6 levels of N trial were not changed significantly after acute RE (Figure 2B). This result was inconsistent with previous studies (Nieman et al., 2004; Kraemer et al., 2014). Similar to the results presented here, a report indicated that acute RE at varying volume loads could not elevate plasma IL-6 levels significantly in human subjects (Raines et al., 2020). Intriguingly, in the present study, RE-induced IL-6 level increases only appeared in HH treatment trial (Figure 2B). IL-6 in circulation after exercise is released mainly from the contracting muscle (Fischer, 2006). Increased IL-6 expression in conjunction with higher levels of muscle damage indicators is usually associated with the disruption of myofibers after exercise (Tomiya et al., 2004). However, in this study, lower levels of CK and Mb in HH trial showed the decreased muscle damage as expected, but unexpectedly IL-6 levels increased. It is speculated that higher release of IL-6 in the HH trial may be explained by activation of the IL-6-related signaling pathway during HH treatment, such as NFκB, Ca^2+^/NFAT, or p38 MAPK. Exercise activates these redox-sensitive intercellular signaling pathways, which in turn cause intramuscular IL-6 release and expression. This signaling pathway activation and IL-6 release during exercise has been shown to be blunted by antioxidant supplementation (Fischer et al., 2004). Therefore, we suggest that HH treatment-induced oxidative stress may activate more IL-6-related signaling pathways and cause more IL-6 release and production. Unfortunately, the related signaling pathway levels in muscle tissue were not measured in this study.

In addition, in this study, elevated IL-6 is proposed as the cause of attenuated muscle damage in HH trial (Figure 2B). IL-6 has been reported as an anti-inflammatory cytokine (Petersen and Pedersen, 2006). For example, anti-IL-6 receptor monoclonal antibody (IL-6r mAb) treatment increased inflammatory response in the mouse model of Duchenne Muscular Dystrophy (mdx; Kostek et al., 2012). Recombinant human IL-6 (rhIL-6) infusion could attenuate *E. coli* lipopolysaccharide endotoxin-induced increase in tumor necrosis factor α (TNF-α) in healthy humans (Starkie et al., 2003). Inflammation plays an important role in muscle damage after an acute bout of RE. Anti-inflammatory treatment, such as Ibuprofen, a non-steroidal anti-inflammatory drug, has been shown to decrease exercise-induced muscle damage (Fraga et al., 2020). The results of this study suggest that the increased IL-6 release in the HH trial will reduce post-exercise inflammation and result in reduced secondary damage of skeletal muscle after RE.

Release of IL-6 is an essential mediator of satellite cell-mediated skeletal muscle regeneration and hypertrophy. IL-6 induced signaling is associated with satellite cell proliferation following acute contraction-induced muscle damage in human subjects (Toth et al., 2011). In cardiotoxin-induced muscle injury/regeneration (Zhang et al., 2013) and compensatory hypertrophy (Serrano et al., 2008) models, myoblast proliferation and muscle regeneration are attenuated in IL-6 knock-out mice compared to wild type mice. The magnitude of IL-6 response after acute resistance is positively correlated with chronic changes in muscle fiber cross-sectional area (CSA) following 16 weeks of resistance training (Mitchell et al., 2013). Accordingly, we hypothesize higher post-exercise IL-6 response induced by HH preconditioning may accelerate skeletal muscle adaptation after chronic resistance training. However, long-term adaptation to chronic HH preconditioning combined with resistance training was not investigated in the present study and remains to be explored.

In the present study, our data show that isokinetic muscle strength decreased significantly immediate after RE in both trials (Figure 4A), but returned to baseline after 24 h. These results are consistent with a previous study (Paulsen et al., 2014). However, the decreased muscle strength after exercise was similar for N and HH trials, and no significant muscle strength difference was found between HH and N trials. The positive effects of HH preconditioning on muscle damage and soreness were strangely not reflected in the resulting muscle strength. We suggest that the HH preconditioning-induced excessive oxidative stress, evidenced by increased TBARS levels, may be contrary to force generation (Powers and Jackson, 2008). Alternatively, influence by other fatigue-related factors cannot be excluded, such as levels of phosphocreatine or glycogen.

Post-exercise circulating cortisol and testosterone levels indicated catabolism and anabolism balance, respectively. Cortisol and testosterone levels often increased significantly immediately after strenuous exercise (Hug et al., 2003). However, in this study, the data showed that when compared to baseline cortisol and testosterone levels do not change significantly immediately after RE in both trials (Figure 3). Cortisol and testosterone levels in blood have large diurnal variation, decreasing from morning to evening (Hayes et al., 2010). Because the time gap between the baseline (before preconditioning) and 0 (immediately after exercise) points is about 2.5 h, we suggest that exercise-induced cortisol and testosterone variations were hidden by larger changes within this period because of the diurnal cycle. In addition, testosterone is a signal for muscle protein synthesis, resulting in muscle growth and other adaptations to resistance training (Hayes et al., 2010). In this study, testosterone levels of HH trial marginally (*p* = 0.062 by Wilcoxon signed rank test) decreased 24 h after exercise (Figure 3B), indicating reduced anabolic signaling. Consequently, we cannot exclude the possibility of decreased training adaptation after chronic resistance training with HH preconditioning.

The underlying mechanism for this HH preconditioning benefit remains unclear. Similar to the hormesis effect, appropriate stressor pre-exposure can result in reduced muscle damage during exercise. For example, preconditioning using hyperthermia (Nosaka et al., 2007), ischemia (Franz et al., 2018), or light exercise (Nausheen et al., 2017) have been shown to reduce RE-induced muscle damage and inflammation. In a previous cell culture study, intermittent hypoxia–hyperoxia treatment elevated oxidative stress production in human airway smooth muscle cells (Bartman et al., 2021). Chronic intermittent hypoxia–hyperoxia also increased oxidative damage in lung tissue of neonatal rodents (Dylag et al., 2017; Mohamed et al., 2020). Thus, we hypothesize that slight oxidative stress induced during HH preconditioning may be responsible for attenuating the subsequent RE-induced muscle damage. In addition, an increased antioxidant protein expression was induced by repeated HH treatment in murine cells (Wohlrab et al., 2021), which might improve the function of the oxidative defense systems. Consequently, we speculate that HH-induced oxidative stress might activate the redox-sensitive signaling system and then enhance the antioxidant defense system. Furthermore, in order to optimize the favorable effect of HH preconditioning, the exposure time, oxygen concentration, and numbers of repetitions of HH intervention could be modified in future research.

The purpose of this study was to analyze the protective effects of acute repeated HH preconditioning on RE-induced muscle damage. The results are ambivalent because a favorable effect of HH treatment on muscle damage and soreness markers was observed, but muscle performance was not significantly affected by the different protocols. It appears that the degree of muscle damage reduced by HH treatment is not enough to influence exercise performance. Muscle damage is induced during sports training and competition and influences training quality and exercise performance (Smith, 1992). In conclusion, we suggest that acute repeated HH treatment has the potential to be developed and optimized as a physical intervention strategy for sports training and competition to prevent muscle damage. The future studies should focus on underlying mechanisms and impact in chronic training applications.

## Data Availability Statement

The raw data supporting the conclusions of this article will be made available by the authors, without undue reservation.

## Ethics Statement

The procedure of this study was reviewed and approved by the University of Taipei Institutional Review Board, Taipei, Republic of China (Taiwan; Approval number: IRB-2016-052).

## Author Contributions

P-WC contributed by recruiting subjects, collecting data, and analyzing statistics. C-CH, L-FL, and C-PC contributed by recruiting subjects and collecting data. S-HY contributed by conceiving the study, interpreting the results, and writing the manuscript. All authors contributed to the article and approved the submitted version.

## Funding

The authors acknowledge financial support from the Ministry of Science and Technology (Grant number: 106-2410-H-197-003-), Republic of China (Taiwan).

## Conflict of Interest

The authors declare that this study does not present them with any conflict of interest resulting from their commercial or financial relationships.

## Publisher’s Note

All claims expressed in this article are solely those of the authors and do not necessarily represent those of their affiliated organizations, or those of the publisher, the editors and the reviewers. Any product that may be evaluated in this article, or claim that may be made by its manufacturer, is not guaranteed or endorsed by the publisher.

## Footnotes

^1^ https://www.stat.ubc.ca/~rollin/stats/ssize/n2.html
